# To Flex or Rest: Does Adding No-Load Isometric Actions to the Inter-Set Rest Period in Resistance Training Enhance Muscular Adaptations? A Randomized-Controlled Trial

**DOI:** 10.3389/fphys.2019.01571

**Published:** 2020-01-15

**Authors:** Brad J. Schoenfeld, Jozo Grgic, Bret Contreras, Kenneth Delcastillo, Andrew Alto, Cody Haun, Eduardo O. De Souza, Andrew D. Vigotsky

**Affiliations:** ^1^Department of Health Sciences, CUNY Lehman College, New York City, NY, United States; ^2^Institute for Health and Sport, Victoria University, Melbourne, VIC, Australia; ^3^Sport Performance Research Institute, Auckland University of Technology, Auckland, New Zealand; ^4^Department of Exercise Science, LaGrange College, LaGrange, GA, United States; ^5^Department of Health Sciences and Human Performance, The University of Tampa, Tampa, FL, United States; ^6^Department of Biomedical Engineering, Northwestern University, Evanston, IL, United States; ^7^Department of Statistics, Northwestern University, Evanston, IL, United States

**Keywords:** rest interval, active rest, training methods, hypertrophy, strength, muscular adaptations

## Abstract

We aimed to investigate the effects of resistance training (RT) combined with no-load isometric actions (iso-holds) during the inter-set recovery period versus RT that involves passive inter-set rest on muscular strength, muscular hypertrophy, and muscular endurance in resistance-trained men. Twenty-seven resistance-trained male volunteers were randomly assigned to either a traditional group (TRAD) that performed a hypertrophy-oriented RT routine with the rest intervals spent passively (*n* = 13) or to a group that supplemented traditional RT with iso-holds (ISO) for the working muscle group between each set (*n* = 14). Training for both routines consisted of three weekly sessions performed for 8 weeks. Three sets of 8–12 repetitions were performed per exercise. A 2-min rest interval was afforded between sets; the ISO group performed iso-holds for the first 30 s of each rest interval and then recovered for the final 90 s. Maximal strength was assessed using the one repetition maximum (1RM) tests in the leg press and bench press. Upper-body muscle endurance was assessed by performing the bench press to failure at 50% of 1RM. Muscle thickness (MT) of the elbow flexors, elbow extensors, mid-thigh, and lateral thigh was assessed using B-mode ultrasound. Results indicated a favorable effect of ISO on MT in the mid-thigh. Alternatively, there was a possible detrimental effect for ISO on leg press strength. No other notable differences were seen between conditions. In conclusion, the use of inter-set iso-holds may be a time-efficient strategy to enhance development of the quadriceps femoris; conversely, it may be detrimental to maximizing lower body strength.

## Introduction

Resistance training (RT) is a popular mode of physical exercise among both the general population and athletes (American College of Sports Medicine, 2009). An RT session involves intermittent bouts of work and rest. The rest interval can be operationally defined as the time taken between sets. Traditionally, rest intervals are spent passively (i.e., without any additional physical activity) and therefore, most of the current recommendations for rest intervals exclusively focus on its optimal duration (American College of Sports Medicine, 2009; Grgic et al., 2018).

Mohamad et al. (2012) highlighted that in an RT session comprising six to eight exercises performed for three to four sets with a rest interval of 60–90 s, the total amount of time spent in rest is ∼24–40 min per session. Given the amount of time allocated to rest in a given RT session, and considering the importance of time efficiency in promoting adherence to RT (Morgan et al., 2016), there is considerable interest in finding strategies that make effective use of the time spent during the rest period. Improvements in efficiency can be achieved either by reducing session duration while achieving similar (or better) results or by maintaining session duration while enhancing results.

Several authors have investigated the effects of inter-set strategies that might hasten between-set recovery and enhance performance on subsequent sets. Hannie et al. (1995) reported that the inclusion of aerobic exercise (as compared to passive rest) during the inter-set period enhances the recovery of maximal voluntary isometric actions (MVC) and increases the total number of repetitions performed in the bench press exercise. Other inter-set strategies previously investigated include dynamic and static stretching, heating and cooling, and foam rolling (Garcia-Lopez et al., 2010; Kwon et al., 2015; Monteiro and Neto, 2016). While there is evidence suggesting that the inclusion of some of these inter-set strategies might enhance performance in resistance exercise, the majority of the current studies examined only their acute effects. Therefore, it remains unclear if employing a given inter-set strategy might also impact long-term adaptations to RT such as muscular strength, muscular hypertrophy, and muscular endurance.

Bodybuilders frequently use posing as a strategy to make their muscles appear as large and defined as possible (Rossow et al., 2013). Posing practice typically involves repeated sustained no-load isometric actions (a.k.a. iso-holds) of major muscle groups for 30–60 s (Rossow et al., 2013). The champion bodybuilder Arnold Schwarzenegger is quoted as saying: “A basic physique is developed by training, but posing adds sharpness and quality” (Schwarzenegger and Dobbins, 1998). This raises the possibility that adding bouts of iso-holds to traditional RT programs may help to enhance muscular adaptations. The effects of iso-holds in the inter-set period on muscular strength and hypertrophy are as yet unexplored. Based on the results from related studies, it can be hypothesized that this strategy might be beneficial for enhancing these adaptations. In a within-subject study design by Counts et al. (2016), 13 untrained participants completed 18 sessions of the unilateral elbow flexion exercise. Each participant trained the elbow flexor of one arm for four sets with 20 repetitions without any external load (i.e., no-load training). The other arm performed 8–12 repetitions with 70% of one repetition maximum (1RM) for four sets in each training session. The acute findings of this study show that both types of training had a similar effect on muscle swelling and the reduction of strength from pre to post-exercise. More interestingly, the longitudinal pre-to-post intervention data from this study indicated that both training conditions had a similar effect on increasing elbow flexor thickness, while the 70% 1RM training condition resulted in greater 1RM strength increases in elbow flexion exercise (+3 versus +1 kg).

Another study by Maeo et al. (2014) allocated nine untrained men to a training group that performed MVCs by simultaneously contracting the elbow flexors and elbow extensors at 90° of the elbow joint without any external resistance for 12 weeks, three times per week. The training program consisted of 4-s MVCs followed by 4 s of relaxation performed for a total of 10 times in each of the five sets. An additional seven participants refrained from training and served as controls. Results indicated that isometric actions were sufficient to yield muscle thickness (MT) improvements of the elbow flexors and elbow extensors (+4% for both) relative to the control group. Furthermore, the training group markedly enhanced the MVC torque of the elbow flexors (+15%) and extensors (+46%) relative to the control group.

While the results of Maeo et al. (2014) and Counts et al. (2016) provide evidence that no-load training *per se* can promote muscular adaptations in untrained individuals, neither study examined if combining iso-holds with traditional RT may have an additive effect that further augments strength and hypertrophy compared to resistance exercise conducted with passive inter-set rest. Moreover, the effects of no-load contractions in individuals with previous RT experience remain unclear.

Given the current gaps in the literature, the present study aimed to compare the effects of traditional RT combined with iso-holds during the inter-set period versus RT that involves passive inter-set rest on muscular strength, muscular hypertrophy, and muscular endurance in resistance-trained men. Based on the previously discussed observations (Maeo et al., 2014; Counts et al., 2016), we hypothesized that the group performing iso-holds during the inter-set period (as compared to the group training with passive inter-set rest periods) would achieve greater increases in muscle size, strength, and endurance.

## Materials and Methods

### Subjects

Subjects were 35 male volunteers recruited from a university population. This sample size was justified by *a priori* precision analysis for the minimum detectable change at the 90% level (MDC_90%_) for biceps thickness (i.e., SEM × *z*_0.05_ = 1.04 × 1.64 = 1.71 mm), such that the compatibility interval (CI) of the between-group effect would be approximately ± MDC_90%_. Based on data from previous research (Schoenfeld et al., 2016b, 2018), along with their sampling distributions, Monte Carlo simulation was used to generate 90% CI widths for 5000 random samples of each sample size. To ensure a conservative estimate, as literature values may not be extrapolatable, the sum of each simulated sample size’s 90% CI’s mean *and* SD was used, and the smallest sample that exceeded MDC_90%_ was chosen; that is, 16 participants per group (1:1 allocation ratio). Additional participants were recruited to account for the possibility of dropout. To qualify for inclusion in the study, the subjects were required to be: (a) between the ages of 18 and 35 years; (b) free from existing cardiorespiratory or musculoskeletal disorders; (c) self-reported as free from consumption of anabolic steroids or any other legal or illegal agents known to increase muscle size currently and for the previous year; and (d) considered as resistance-trained, defined as consistently lifting weights at least three times per week (on most weeks) for at least 1 year.

**FIGURE 1:**
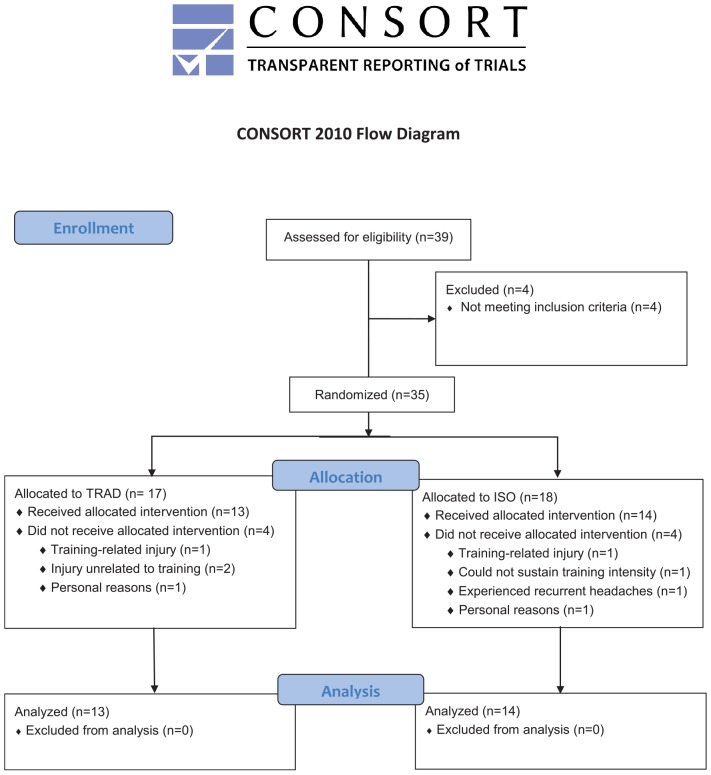
CONSORT flow diagram.

Participants were randomly assigned to one of two experimental, parallel groups: a traditional group (TRAD) that performed a traditional hypertrophy-oriented RT routine with passive inter-set rest (*n* = 17) or a group that supplemented traditional RT with no-load isometric actions (ISO) for the working muscle group during the inter-set recovery period (*n* = 18). Randomization was carried out using online software^1^. Approval for the study was obtained from the college Institutional Review Board. Informed consent was obtained from all participants prior to beginning the study. A CONSORT flow diagram of the study is presented in Figure 1.

### Resistance Training Procedures

The RT protocol consisted of the following six exercises per session targeting major muscle groups of the body: flat barbell bench press, barbell military press, wide grip lat pulldown, seated cable row, barbell back squat, and machine leg press. These exercises were chosen based on their common inclusion in bodybuilding- and strength-type RT programs (Baechle and Earle, 2008; Coburn and Malek, 2011). Subjects were instructed to refrain from performing any additional resistance-type or high-intensity anaerobic training for the duration of the study.

Training for both routines consisted of three weekly sessions for 8 weeks. Three sets of 8–12 repetition maximum (RM) were performed per exercise. All sets were carried out to the point of momentary concentric muscular failure (i.e., the inability to perform another concentric repetition while maintaining proper form). Training to muscle failure in trained individuals has been shown to impair recovery for 24–48 h post-exercise (Moran-Navarro et al., 2017). Therefore, training sessions were carried out on non-consecutive days with at least 48 h between sessions to facilitate inter-session recovery. The cadence of repetitions was carried out in a controlled fashion, with a concentric action of approximately 1 s and an eccentric action of approximately 2 s. Subjects were afforded 2 min rest between sets whereby they either sat or stood relatively motionless between sets. The load was subjectively adjusted for each exercise as needed on successive sets to ensure that subjects achieved failure in the target repetition range. All routines were directly supervised by the research team to monitor the proper performance of the respective routines and ensure participant safety. Attempts were made to progressively increase the loads lifted each week within the confines of maintaining the target repetition range. Prior to training, subjects underwent 10RM testing to determine individual initial training loads for each exercise. The RM testing was consistent with recognized guidelines as established by the National Strength and Conditioning Association (Baechle and Earle, 2008).

The ISO group performed each routine as described above and then supplemented training with the performance of no-load isometric actions for the elbow flexors, elbow extensors, or quadriceps femoris immediately following performance of each set. Specifically, bilateral isometric actions for the elbow flexors followed performance of the lat pulldown and seated row; isometric actions for the elbow extensors followed performance of the flat barbell press and barbell military press; and isometric actions for the quadriceps femoris followed performance of the barbell back squat and machine leg press. Subjects held the isometric actions for 30 s (Rossow et al., 2013), and then rested for the remainder of the inter-set rest period. For the elbow flexors, subjects were instructed to keep the upper arms pressed to the sides and flex the elbow as far as comfortably possible; for the elbow extensors, subjects were instructed to keep the upper arms pressed to the sides and extend the elbow as far as comfortably possible, and for the quadriceps femoris subjects were seated and extended the knees as far as comfortably possible. During all isometric holds, the research team prodded subjects to “squeeze” (i.e., contract) the muscle as hard as possible for the 30-s duration of each rest period.

### Dietary Adherence

To avoid potential dietary confounding of results, subjects were advised to maintain their customary nutritional regimen and to avoid taking any supplements other than that provided in the course of the study. Dietary adherence was assessed by self-reported food records using MyFitnessPal.com^2^, which were collected twice during the study: 1 week before the first training session (i.e., baseline) and during the final week of the training protocol (Burke et al., 2005). Subjects were instructed on how to properly record all food items and their respective portion sizes consumed for the designated period of interest. Each item of food was individually entered into the program, and the program provided relevant information as to total energy consumption, as well as the amount of energy derived from proteins, fats, and carbohydrates for each time period analyzed. To help ensure that dietary protein needs were met, subjects were given a supplement on training days containing 24 g protein and 1 g carbohydrate (Iso100 Hydrolyzed Whey Protein Isolate, Dymatize Nutrition, Dallas, TX, United States) that was consumed under the supervision of the research team to ensure compliance.

### Measurements

#### Anthropometry

Subjects were told to refrain from eating for 12 h prior to testing, eliminate alcohol consumption for 24 h, abstain from strenuous exercise for 24 h, and void immediately before the test. Participants’ height was measured using a stadiometer and body mass was assessed using a calibrated scale.

#### Muscle Thickness

Ultrasound imaging was used to obtain measurements of MT. The reliability and validity of ultrasound in determining MT has been reported to be very high when compared to the “gold standard” magnetic resonance imaging (Reeves et al., 2004). The lead researcher, a trained ultrasound technician, performed all testing using a B-mode ultrasound imaging unit (Model E1, SonoScape, Co., Ltd., Shenzhen, China). The technician applied a water-soluble transmission gel (Aquasonic 100 Ultrasound Transmission gel, Parker Laboratories Inc., Fairfield, NJ, United States) to each measurement site, and a 4–12 MHz linear array ultrasound probe was placed perpendicular to the tissue interface without depressing the skin (Schoenfeld et al., 2019). When the quality of the image was deemed to be satisfactory, the technician saved the image to a hard drive and obtained MT dimensions by measuring the distance from the subcutaneous adipose tissue–muscle interface to the muscle–bone interface. Measurements were taken on the right side of the body at four sites: (1) elbow flexors, (2) elbow extensors, (3) mid-thigh (a composite of the rectus femoris and vastus intermedius), and (4) lateral thigh (a composite of the vastus lateralis and vastus intermedius). For the anterior and posterior upper arm, measurements were obtained 60% distal between the lateral epicondyle of the humerus and the acromion process of the scapula; mid- and lateral thigh measurements were obtained 50% between the lateral condyle of the femur and greater trochanter for the quadriceps femoris. To ensure that swelling in the muscles from training did not obscure MT results, images were obtained at least 48 h after the training sessions, both in the pre- and post-study assessment. This is consistent with research showing that acute increases in MT return to baseline within 48 h following an RT session (Ogasawara et al., 2012) and that muscle damage is minimal after repeated exposure to the same exercise stimulus over time (Damas et al., 2016; Biazon et al., 2019). To further ensure accuracy of measurements, three images were obtained for each site and then averaged to obtain a final value. The test–retest intraclass correlation coefficient (ICC) from our lab for thickness measurement of the elbow flexors, elbow extensors, mid-thigh, and lateral thigh are 0.942, 0.933, 0.957, and 0.941, respectively; the coefficients of variation (CV) for these measures are 2.2, 2.1, 1.6, and 1.4%, respectively.

### Maximal Strength Assessments

#### Muscle Strength

Upper and lower body strength were assessed by 1RM testing in the 45^°^ angled leg press (1RM_LEGPRESS_) and the bench press (1RM_BENCH_) exercises. Subjects reported to the lab having refrained from any exercise other than activities of daily living for at least 48 h prior to baseline testing and at least 48 h prior to testing at the conclusion of the study. RM testing was consistent with recognized guidelines as established by the National Strength and Conditioning Association (Baechle and Earle, 2008). In brief, subjects performed a general warm-up prior to testing consisting of light cardiovascular exercise lasting approximately 5–10 min. Next, a specific warm-up set of the given exercise of five repetitions was performed at ∼50% 1RM followed by one to two sets of two to three repetitions at a load corresponding to ∼60–80% 1RM. Subjects then performed sets of one repetition of increasing weight for 1RM determination. Three to 5 min rest was afforded between each successive attempt. All 1RM determinations were made within five attempts.

Testing for the 1RM_BENCH_ was carried out using a barbell in a bench press safety rack. Successful performance was determined as follows: Subjects laid supine on the bench, assumed a five-point body contact position (head, upper back, and buttocks firmly on the bench with both feet flat on the floor), and grasped the bar at a comfortable distance. Subjects received assistance removing the barbell from the rack, bringing the weight down until it touched the chest without bouncing, and then executed a full lock-out. All testing sessions were supervised by two research assistants to achieve a consensus for success on each attempt. The test–retest ICC from our lab for the 1RM_BENCH_ is 0.996 with a CV of 1.7%.

Testing for 1RM_LEGPRESS_ was carried out on a plate-loaded angled leg press (Life Fitness, Westport, CT, United States). Successful performance was determined as follows: subjects sat upright on the angled leg-press machine, placed their feet on the footplate with a hip-width stance, straightened their legs with toes angled 10^°^ outward, and then unlocked the carriage-release bars located on the sides of the machine. Keeping their backs pressed firmly against the padded seat, subjects lowered the carriage by bringing the knees toward the chest until the thighs and lower leg formed a 90^°^ knee angle without bouncing at the bottom. The weight then was pushed up in a controlled fashion until the knees were fully extended. The test–retest ICC from our lab for the 1RM_LEGPRESS_ is 0.929 with a CV of 5.3%.

#### Muscle Endurance

Upper-body muscular endurance was assessed by performing the bench press using 50% of the subject’s initial 1RM in the bench press (50%_BENCH_) for as many repetitions as possible to muscular failure with proper form. Successful performance was achieved if the subject displayed a five-point body contact position (head, upper back, and buttocks firmly on the bench with both feet flat on the floor) and executed a full lock-out on all repetitions. Muscular endurance testing was carried out after assessment of muscular strength to minimize effects of metabolic stress potentially interfering with performance of the latter. Moreover, muscle endurance testing was performed after the 1RM_LEGPRESS_—approximately ½ h after the 1RM_BENCH_—to allow for adequate recovery between the 1RM_BENCH_ and 50%_BENCH_ (Schoenfeld et al., 2015, 2016a, 2019). The test–retest ICC from our lab for the 50% _BENCH_ is 0.903 with a CV of 3.1%.

### Statistical Analyses

Data were analyzed in R (version 3.5.3) (R: A language and environment for statistical computing, 2019). Our analytical approach may be considered unconventional relative to commonly applied methods in sports and exercise science, so we present our rationale for each analytical decision. In line with recommendations from the biostatistics, methodology, and reporting guidelines literatures, (1) inferential statistics on baseline measures and demographics were not calculated given that the null hypothesis is necessarily true due to randomization, we are not interested in making population inferences about baseline characteristics, and we control for baseline values in our analyses (Altman, 1985; Senn, 1994; Moher et al., 2010); and (2) within-group inferential statistics were not calculated since they do not address our between-group research question and can be inferentially misleading (Matthews and Altman, 1996; Moher et al., 2010; Bland and Altman, 2011, 2015).

To answer our research question, the effect of group (ISO versus TRAD) on each outcome variable was estimated using linear regression with pre-intervention score included as a nuisance variable (Vickers and Altman, 2001), enabling us to control for regression to the mean and baseline differences. All outcomes were modeled using ordinary least squares, except for muscle endurance, which was modeled using Poisson regression since the data are counts (non-negative integers rather than continuous; number of repetitions reported as log counts). Model residuals were qualitatively examined for structure and heteroscedasticity, but normality was not checked since CIs were calculated non-parametrically via the bootstrap. We computed 90% CIs of the adjusted effects using the bias-corrected and accelerated bootstrap with 1000 replicates (Davison and Hinkley, 1997; Efron, 2012; Canty and Ripley, 2019).

When drawing inferences, we were interested in the magnitude and uncertainty of each outcome, whether it be close to zero or otherwise, without falsely dichotomizing the existence of an effect. Thus, we do not employ traditional null hypothesis significance testing, which has been extensively criticized for its use in the biomedical and social sciences (Amrhein et al., 2019; McShane et al., 2019). Instead, we decided *a priori* to draw inferences via an estimation approach (Gardner and Altman, 1986). In doing so, we consider the implications of all results that are compatible with these data, from the lower limit to the upper limit of the CI, with the greatest interpretive emphasis placed on the point estimate. Plots were generated using augmented partial residuals and the *dabestr* package (Ho et al., 2019).

To ensure robustness of the findings, sensitivity analyses were performed on the primary outcome measures to detect the presence of outliers or individuals that may have inflated or attenuated the observed effects and their uncertainty–participants who strongly influence the outcomes would affect the point estimate (in either direction) and increase the standard error due to the increase in heterogeneity. To accomplish this, we performed leave-one-out analyses, in which each participant was removed from the analysis and the analysis was repeated as if that participant was not in the study. The resulting effects and their standard errors were examined qualitatively.

Secondary analyses were performed on the volume load and nutrition data. Volume load was determined by multiplying total repetitions per set by the load lifted for each exercise. Volume loads were summed within a day and averaged across days, so as not to prevent any bias that may arise due to missing sessions. This process was repeated for the first and last 3 days of each participant’s attendance, in addition to the entire study. Average volume load per exercise per day was compared using descriptive statistics (mean ± SD). Nutrition data were analyzed similarly to the MT and strength data; that is, using multiple regression with group dummy-coded and pre-intervention nutrition scores as covariates of no interest. The results of these secondary analyses are presented using mean adjusted effects and their standard errors.

## Results

Eight subjects withdrew from the study for the following reasons: Injury not related to training (two subjects); minor training-related injury (two subjects); could not sustain intensity of program (one subject); experienced headaches during training (one subject); personal reasons (two subjects). Thus, a total of 27 subjects completed the study (13 in TRAD; 14 in ISO). The observed precision for the biceps brachii thickness effect was lower than the target precision (1.45 < 1.71 mm), indicating that the conservative approach to the sample size calculation for precision was successful, even in light of dropouts. Overall attendance was satisfactory (Gentil and Bottaro, 2013), with subjects completing 92% of sessions (91% in TRAD; 93% in ISO).

Baseline measures for participants in each group are presented in Table 1. Descriptive statistics of the within-group pre-, post-, and change-scores for all primary outcomes, in addition to between-group adjusted effects and their CIs, can be found in Table 2.

**TABLE 1:** Participant demographics.

**Variable**	**TRAD**	**ISO**
Height (cm)	176.8 ± 4.5	174.0 ± 4.5
Weight (kg)	81.9 ± 14.6	87.0 ± 17.1
Age (years)	22.0 ± 3.8	24.2 ± 3.8
Training experience (years)	3.3 ± 2.5	3.4 ± 2.4

*All values are reported as mean ± SD.*

**TABLE 2:** Experimental outcomes.

	**TRAD**			**ISO**			**Between-group**
	**Pre**	**Post**	**Delta**	**Pre**	**Post**	**Delta**	**Estimate (90% CI)**
Elbow flexors (mm)	40 ± 7	43 ± 8	2.9 ± 1.7	42 ± 7	46 ± 6	3.5 ± 2.5	0.7(−0.8,2.1)
Elbow extensors (mm)	41 ± 7	43 ± 8	1.6 ± 2	44 ± 8	46 ± 10	2.3 ± 3.6	0.3(−1.5,2.2)
Mid-thigh (mm)	58 ± 11	61 ± 9	2.8 ± 4.4	60 ± 9	65 ± 9	4.8 ± 3.6	2.3(−0.8,4.3)
Lateral thigh (mm)	55 ± 9	60 ± 8	4.5 ± 3.4	58 ± 9	62 ± 9	4.1 ± 3.4	−0.1(−2.2,2.0)
1RM bench press (kg)	93 ± 26	102 ± 27	8.9 ± 7.8	94 ± 21	104 ± 25	9.4 ± 7.8	0.5(−4.3,5.6)
1RM leg press (kg)	310 ± 110	407 ± 99	97 ± 57	339 ± 76	412 ± 100	73 ± 42	−21.7(−54.5,8.5)
Endurance (repetitions)^∗^	27 ± 7	30 ± 9	4 ± 5	26.5 ± 3.5	31.5 ± 6.5	3.5 ± 8.5	0.06(−0.02,0.2)

*^∗^Data are presented as median ± interquartile range and log counts for the between-group effect. 1RM = one repetition maximum; TRAD = group performing resistance training with passive inter-set rest; ISO = group performing resistance training with 30-s isometric actions during the inter-set rest period; CI = compatibility intervals.*

### Muscle Thickness

**FIGURE 2:**
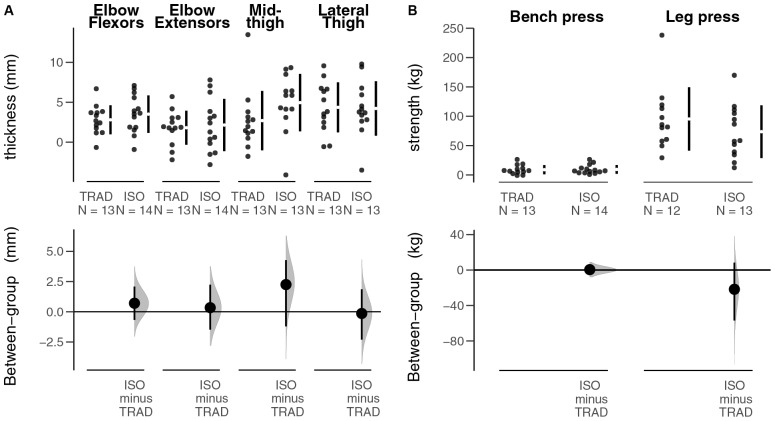
Muscle thickness and strength outcomes. Top plots represent adjusted individual changes, and bottom plots represent adjusted between-group effects. **(A)** Muscle thickness outcomes of the biceps brachii, triceps brachii, rectus femoris, and vastus lateralis. **(B)** Strength outcomes for both bench press and leg press 1RM. Error bars indicate 90% CIs, and distributions represent the bias-corrected and accelerated bootstrap distribution of the adjusted group effect.

Figure 2A shows the scatterplot of individual results for MT. For the elbow flexors, elbow extensors, and lateral thigh, the adjusted estimates of the difference in MT changes between groups were miniscule (≤0.7 mm in either direction). Moreover, CIs of these adjusted estimates did not encapsulate remarkably favorable estimates for either group (≤2.2 mm in either direction). The point estimate of the adjusted effect of mid-thigh thickness changes favored the ISO group, but only modestly so (2.3 mm). The data are compatible with both a negligible change in favor of the TRAD group and an appreciable change in favor of the ISO group (−0.8–4.3 mm). Leave-one-out sensitivity analyses indicated that data from one individual markedly increased mid-thigh growth in TRAD while that from another individual markedly attenuated growth in ISO; removal of each of these data separately increased the point estimate to 3.2 mm favoring ISO and narrowed the width of CI (0.6–4.8 mm) such that all values of the CI favored ISO, even if modestly so (Supplementary Figure S1).

### Muscular Strength

Figure 2B shows the scatterplot of individual results for muscular strength. The adjusted effect for the 1RM_BENCH_ changes was small (0.5 kg), and its CI did not reveal estimates that were especially favorable in either direction. For the 1RM_LEGPRESS_, two subjects (one in each group) progressed to the point that their post-study 1RM exceeded the limits of the leg press apparatus; thus, results on this outcome were analyzed from 12 subjects in TRAD and 13 subjects in ISO. The adjusted effect for the 1RM_LEGPRESS_ changes favored TRAD (by 21.7 kg), with CI estimates ranging from an 8.5 kg benefit for the ISO group to a 54.5 kg benefit for the TRAD group. Leave-one-out sensitivity analysis suggests that the leg press effect was largely driven by a single individual; after removing his data, the effect was halved [leave-one-out = −10.6 (−37.9–13.7) kg] (Supplementary Figure S2).

In addition to performing analyses on strength outcomes using absolute loads, we also carried out *post hoc* analyses using loads normalized to body mass at baseline. The results of these *post hoc* analyses using normalized loads are similar to those using the absolute loads and can be found in Supplementary Table S1.

### Muscular Endurance

The adjusted effect for the 50%_BENCH_ as determined by the log number of repetitions was 0.06 in favor of the ISO group, with tight CIs around this estimate (−0.02–0.2). Exponentiated, this estimate suggests that those in the ISO group increased their repetition count by 6.5% more than those in the TRAD group, with a CI containing values ranging from 2% greater improvement in TRAD to 22% greater improvement in ISO.

### Volume Load

Descriptive results for volume load changes over the course of the study are presented in Table 3. Total volume load across the study period was relatively similar. However, the ability to increase volume load from the first to last week of the study for a given exercise varied by condition, with some exercises favoring increases for TRAD and others favoring ISO.

**TABLE 3:** Volume load effects.

	**Average of first three sessions**		**Average of last three sessions**		**Average over entire study**	
	**TRAD**	**ISO**	**TRAD**	**ISO**	**TRAD**	**ISO**
Bench	1840 ± 487	1789 ± 440	1877 ± 451	2074 ± 725	1841 ± 483	1861 ± 443
Lat pulldown	1717 ± 382	1596 ± 331	1994 ± 629	1759 ± 339	1805 ± 269	1704 ± 287
Leg press	5607 ± 1680	6557 ± 2335	8180 ± 2764	8286 ± 2418	7267 ± 2336	7617 ± 2383
Military press	886 ± 246	930 ± 228	1029 ± 280	1087 ± 268	991 ± 267	1033 ± 237
Row	1487 ± 325	1432 ± 296	1605 ± 342	1639 ± 413	1557 ± 335	1645 ± 375
Squat	2081 ± 849	2324 ± 388	2257 ± 536	2636 ± 605	2270 ± 587	2481 ± 535

*All values are in kg and reported as mean ± SD. TRAD = group performing resistance training with passive inter-set rest; ISO = group performing resistance training with 30-s isometric actions during the inter-set rest period*

### Nutritional Intake

The results for self-reported nutritional intake during the first and last week of the study are presented in Table 4. Energy intake was modestly higher in TRAD compared to ISO, with the additional calories attributed to a greater carbohydrate consumption. Given the relatively high discrepancies found between self-reported nutritional intake versus actual intake (Mertz et al., 1991), and given the relatively modest differences reported herein, these findings are of questionable significance as to the studied outcomes.

**TABLE 4:** Nutritional intake.

	**TRAD**			**ISO**			**Between-group**
	**First week**	**Eighth week**	**Delta**	**First week**	**Eighth week**	**Delta**	**Estimate ± SE**
Calories (kcal)	1855 ± 702	1950 ± 638	94 ± 549	1937 ± 624	1784 ± 575	−153 ± 452	−216 ± 172
Fat (g)	61 ± 23	64 ± 24	3 ± 21	63 ± 33	73 ± 34	9 ± 42	8 ± 11
Carbohydrates (g)	218 ± 85	234 ± 71	16 ± 75	209 ± 103	168 ± 73	−41 ± 105	−63 ± 26
Protein (g)	98 ± 48	98 ± 40	0 ± 49	95 ± 35	103 ± 40	8 ± 37	6 ± 14

*Within-group descriptive statistics are reported as mean ± SD. TRAD = group performing resistance training with passive inter-set rest; ISO = group performing resistance training with 30-s isometric actions during the inter-set rest period; CI = compatibility intervals.*

## Discussion

To our knowledge, this is the first study to investigate the effect of integrating iso-holds into the inter-set period of a traditional RT program. The study produced several novel findings, specifically: (a) ISO showed a potential benefit for enhancing mid-thigh growth, whereas it had no remarkable additive effect on the other muscles studied; (b) ISO did not seem to produce notable additional benefits for upper-body strength and might have impaired lower-body strength gains; and, (c) increases in muscular endurance were similar between the TRAD and ISO groups.

Results for MT showed similar increases for all sites measured except for the mid-thigh, which favored the ISO group. This finding may be explained by the fact that iso-holds for the quadriceps were performed in the seated position with the knees extended—a position similar to the end phase of the leg extension exercise. Consistent with the patterns of excitatory and inhibitory actions to motor nuclei of the hip and knee musculature (Eccles and Lundberg, 1958), there is evidence that isolated knee extension exercise elicits greater electromyography amplitude of the rectus femoris versus combined hip and knee extension, such as in the barbell squat and leg press (Andersen et al., 2006; Ema et al., 2016). These findings are supported by MRI data, whereby contrast shifts (i.e., alterations in signal intensity) indicate preferential rectus femoris activation during open- (e.g., knee extension) versus closed-chain (e.g., squat) exercise (Enocson et al., 2005). Additionally, in contrast to isolated leg extension training interventions (Ema et al., 2013), training regimens employing only squat and passive rest intervals with variation in range of motion have failed to report increases in rectus femoris muscle growth (Kubo et al., 2019). Given that the design of our study included only multi-joint lower-body exercises (squat and leg press), it is conceivable that performing the iso-holds in a position conducive to activating the rectus femoris may have heightened tension to this part of the muscle and thus enhanced its development. While the overall statistical analysis showed differences in mid-thigh thickness to be rather modest between conditions, the results strengthened after LOO adjustment for one of two highly influential participants to the extent that the magnitude of effect was similar to that seen with regimented leg extension training in longitudinal research (Ema et al., 2013). These findings suggest that the implementation of inter-set iso-holds can be a viable strategy to increase rectus femoris size without additional time spent training.

Increases in 1RM_LEGPRESS_ decidedly favored the TRAD group, with the point estimate indicating a 21.7 kg benefit. The findings potentially can be explained, at least in part, by the presence of a statistically influential participant in the TRAD group as determined by leave-one-out sensitivity analysis. Removal of the data from this subject essentially halved the magnitude of effect, which renders the practical meaningfulness of results questionable. It is also possible that alterations in volume load may have played a role in this finding. Specifically, the TRAD group displayed a greater increase in volume load for the leg press from the first to last week of the study compared to the ISO group (47.7 versus 30.0%, respectively). It seems reasonable to speculate that increases in volume load would be driven by increases in strength, which in turn would suggest that the observed discrepancies between conditions may have influenced 1RM_LEGPRESS_ performance over time. However, strength increases were similar between conditions in the 1RM_BENCH_ despite the fact that the ISO group showed a greater increase in bench press volume load across the study period versus the TRAD (15.3 versus 3.5%, respectively), which would seem to refute this hypothesis.

It should be noted that the observed increases in mid-thigh hypertrophy did not translate into greater strength increases. This may be explained, at least in part, by the methods used to assess muscle growth. Specifically, evidence indicates that MT does not show a high correlation with maximal strength (Vigotsky et al., 2018). Moreover, differences in quadriceps hypertrophy were limited to only the mid-thigh at the location of the rectus femoris. Given evidence that the rectus femoris is preferentially involved in single-joint knee extension (Enocson et al., 2005), it can be inferred that increases in its size do not meaningfully contribute to strength increases as measured by multi-joint lower body exercise.

Muscular endurance outcomes slightly favored ISO, but the magnitude of the effects was relatively modest. From a physiological standpoint, it seems intuitive that performing isometric actions immediately after completion of a set would promote the persistent occlusion of vessels and thus heighten the accumulation of metabolites. Accordingly, we had speculated that consistently subjecting muscles to high levels of H+ would enhance the body’s ability to buffer acidosis, as observed when training with high repetitions and short rest intervals (Edge et al., 2006), which conceivably would promote favorable effects on muscle endurance. This seemed to occur only to a limited extent. One possible explanation is that the TRAD condition resulted in substantial increases in H+ accumulation that reached a critical threshold, beyond which minimal further improvements in buffering capacity could be realized. Alternatively, it is possible that the iso-holds did not appreciably increase metabolite buildup over and above what was produced by TRAD. These hypotheses remain speculative as we did not attempt to measure markers of metabolic stress; further study is warranted to determine a mechanistic rationale for this outcome.

Our study had several limitations that should be taken into account when attempting to draw inferences from the data. First, we obtained MT only at the mid-portion of each muscle. While such measures correlate well with MRI-derived assessments of cross-sectional area (Franchi et al., 2018), there is evidence that muscle growth often manifests in a non-uniform manner across the length of a muscle (Ema et al., 2013); thus, we cannot rule out the possibility that hypertrophic changes may have occurred to a greater extent either proximally or distally in one condition versus the other. Second, we attempted to control dietary practices by having subjects fill out 5-day food diaries at the beginning and end of the study in concert with a trained nutritionist. While food diaries are a well-accepted method for estimating nutritional consumption, evidence indicates widespread discrepancies between what is reported and actual consumption (Mertz et al., 1991). It therefore remains possible that despite our efforts, between-group differences in nutritional factors may have confounded results. Third, our sample comprised young resistance-trained men; hence, results cannot necessarily be generalized to other populations including adolescents, women, and older individuals. Further research is warranted to determine whether inter-set iso-holds may confer a benefit in other populations. Fourth, subjects in ISO were instructed to squeeze the muscle has hard as possible, but we did not employ objective measures (e.g., rating of perceived exertion) to attempt to quantify the effort. Future research may benefit from inclusion of objective assessments of the effort employed during performance of iso-holds. Finally, these data do not preclude the possibility that iso-holds employed differently (e.g., following a workout rather than inter-set) could elicit more (or perhaps less) favorable effects on muscular adaptations; this is an area for future study.

## Conclusion

The implementation of iso-holds during the intra-set rest period may elicit a favorable effect on MT in the mid-thigh, at least when only multi-joint exercise is performed to target the quadriceps femoris. Alternatively, iso-holds may blunt improvements in lower body strength. Thus, the use of inter-set iso-holds may be a time-efficient strategy to enhance development of the quadriceps femoris; conversely, it may be detrimental to maximizing lower body strength.

## Practical Applications

Those interested in maximizing muscular hypertrophy can consider employing iso-holds in the inter-set period, as this strategy potentially may help to enhance hypertrophy of the quadriceps without increasing total training duration. Alternatively, for those interested in maximizing gains in muscular strength, the use of iso-holds during the inter-set recovery period confers no noteworthy benefits and should likely be avoided as our data indicate that it might have a detrimental effect on lower-body strength gains, at least in the short-term. It is possible, however, that an eventual delayed training effect for strength could occur from the additional hypertrophy of iso-holds in future training phases. This hypothesis warrants further investigation.

## Data Availability Statement

The datasets generated for this study are available on request to the corresponding author.

## Ethics Statement

The studies involving human participants were reviewed and approved by the City University of New York. The patients/participants provided their written informed consent to participate in this study.

## Author Contributions

BS designed the study, interpreted results, and contributed to writing and revising the manuscript. AV analyzed and interpreted results, and contributed to writing and reviewing the manuscript. JG, BC, KD, AA, CH, and ED interpreted results and contributed to writing and reviewing the manuscript. All authors approved the final manuscript draft.

## Conflict of Interest

The authors declare that the research was conducted in the absence of any commercial or financial relationships that could be construed as a potential conflict of interest.

## Footnotes

1 randomizer.org

2 http://www.myfitnesspal.com
