# Supplementary material for: To Flex or Rest: Does Adding No-Load Isometric Actions to the Inter-Set Rest Period in Resistance Training Enhance Muscular Adaptations? A Randomized-Controlled Trial
Source: Front Physiol. 2020 Jan 15;10:1571. doi: 10.3389/fphys.2019.01571 (PMC6974452; doi:10.3389/fphys.2019.01571)
Supplement: Supplementary file 1 [file Data_Sheet_1.PDF]

Supplemental Results

Normalized strength outcomes

The adjusted effect for the $1RM_{BENCH}$ changes was negligible (0), and its compatibility interval did not reveal estimates that were especially favorable in either direction (Table S1). For the $1RM_{LEGPRESS}$, 2 subjects (1 in each group) progressed to the point that their post-study 1RM exceeded the limits of the leg press apparatus; thus, results on this outcome were analyzed from 12 subjects in TRAD and 13 subjects in ISO. The adjusted effect for the $1RM_{LEGPRESS}$ changes favored TRAD (by 0.36 bodyweights), with CI estimates ranging from a 0.03 bodyweights benefit for the ISO group to a 0.86 bodyweights benefit for the TRAD group (Table S1).

Table S1. Normalized strength outcomes

	TRAD			ISO			**Between-group**
	Pre	Post	Delta	Pre	Post	Delta	Estimate (90% CI)
**1RM Bench press (BWs)**	1.1 ± 0.2	1.2 ± 0.2	0.1 ± 0.1	1.1 ± 0.2	1.2 ± 0.2	0.1 ± 0.1	0 (−0.05, 0.05)
**1RM Leg press (BWs)**	3.8 ± 1.0	5.1 ± 0.9	1.3 ± 0.8	4.0 ± 0.6	4.9 ± 0.8	0.9 ± 0.5	0.36 (−0.86, 0.03)

1RM = one repetition maximum; TRAD = group performing resistance training with passive inter-set rest; ISO = group performing resistance training with 30-second isometric actions during the inter-set rest period; CI = compatibility intervals; BW = bodyweight

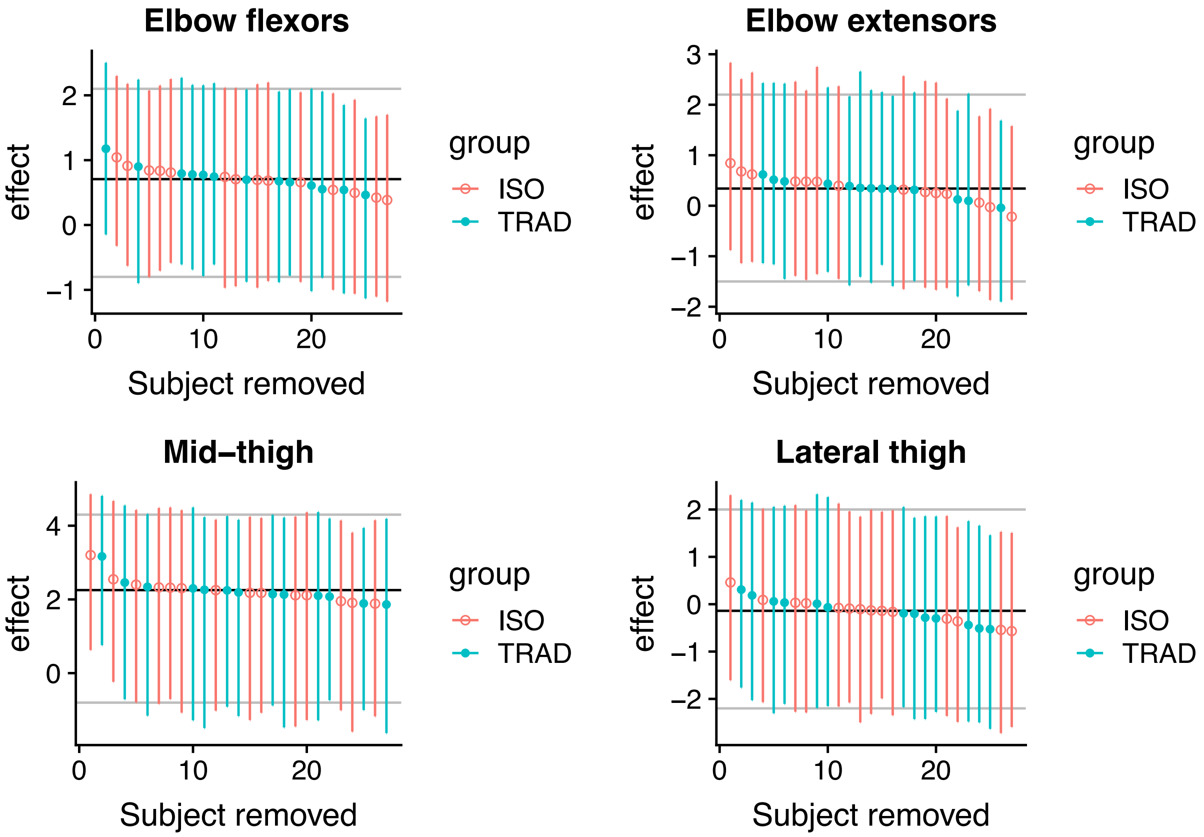

Figure S1. Leave-one-out sensitivity analyses for muscle thickness outcomes. All effects are presented in millimeters of muscle thickness. Black horizontal lines indicate the study-wide effect and grey lines indicate the study-wide 90% CI. Note subjects #1 and #2 drastically affect the estimates and CI's for mid-thigh muscle thickness. CI = compatibility interval.

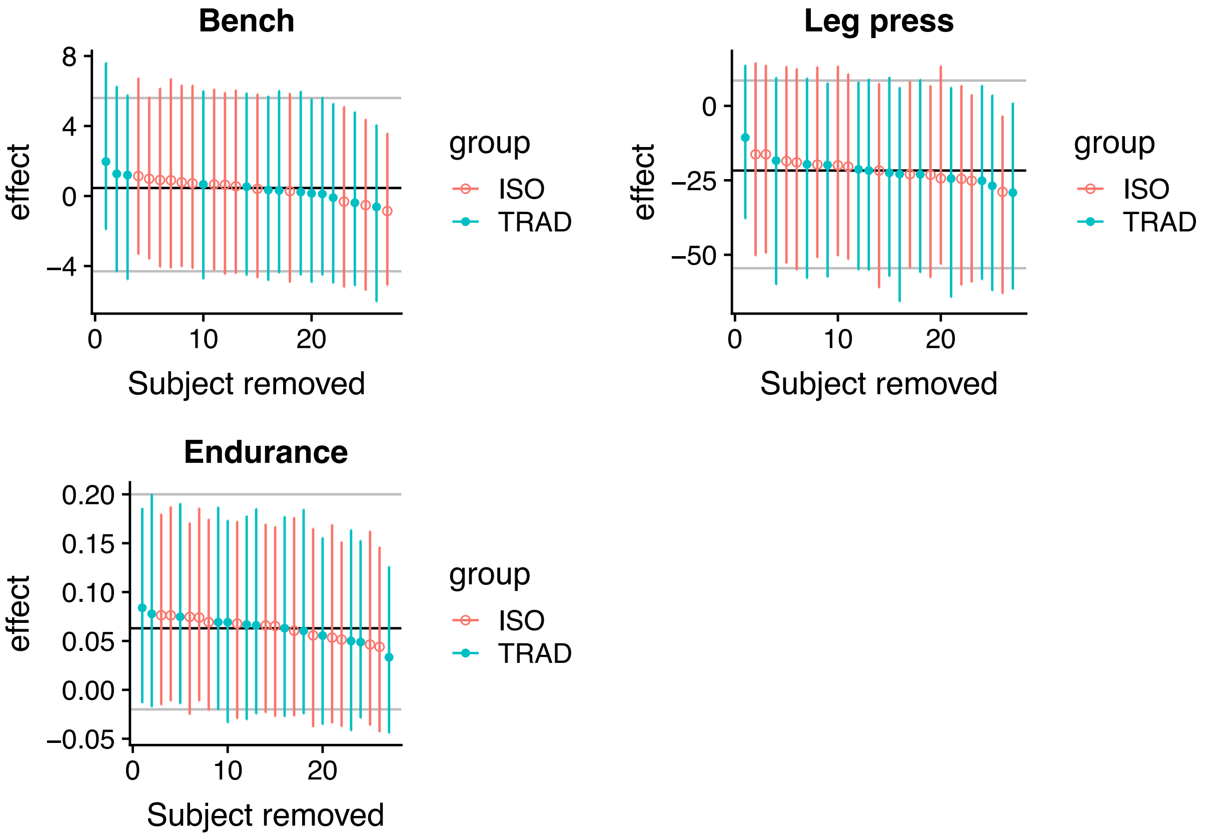

Figure S2. Leave-one-out sensitivity analyses for strength and endurance outcomes. Bench and leg press outcomes are presented as kilograms from the one-repetition maximum test. Endurance is presented as ln(repetitions). Black horizontal lines indicate the study-wide effect and grey lines indicate the study-wide 90% CI. Note that subject #1 in the leg press has a marked effect on the study-wide outcome. CI = compatibility interval.
